# Antifungal Activity of the Enterococcus faecalis Peptide EntV Requires Protease Cleavage and Disulfide Bond Formation

**DOI:** 10.1128/mBio.01334-19

**Published:** 2019-07-02

**Authors:** Armand O. Brown, Carrie E. Graham, Melissa R. Cruz, Kavindra V. Singh, Barbara E. Murray, Michael C. Lorenz, Danielle A. Garsin

**Affiliations:** a Department of Microbiology and Molecular Genetics, The University of Texas Health Science Center at Houston, Houston, Texas, USA; b MD Anderson Cancer Center UTHealth Graduate School of Biomedical Sciences, Houston, Texas, USA; c Division of Infectious Diseases, Department of Internal Medicine, The University of Texas Health Science Center at Houston, Houston, Texas, USA; University of Minnesota Medical School; Nanyang Technological University; University of Kansas

**Keywords:** *Candida*, *Enterococcus*, antifungals, bacteriocins

## Abstract

Enterococcus faecalis, a Gram-positive bacterium, and Candida albicans, a polymorphic fungus, are common constituents of the microbiome as well as increasingly problematic causes of infections. Interestingly, we previously showed that these two species antagonize each other’s virulence and that E. faecalis inhibition of C. albicans was specifically mediated by EntV. EntV is a bacteriocin encoded by the *entV* (*ef1097*) locus that reduces C. albicans virulence and biofilm formation by inhibiting hyphal morphogenesis. In this report, we studied the posttranslational modifications necessary for EntV antifungal activity. First, we show that the E. faecalis secreted enzyme gelatinase (GelE) is responsible for cleaving EntV into its 68-amino-acid, active form and that this process does not require the serine protease SprE. Furthermore, we demonstrate that a disulfide bond that forms within EntV is necessary for antifungal activity. Abrogating this bond by chemical treatment or genetic modification rendered EntV inactive against C. albicans. Moreover, we identified the likely catalyst of this disulfide bond, a previously uncharacterized thioredoxin within the E. faecalis genome called DsbA. Loss of DsbA, or disruption of its redox-active cysteines, resulted in loss of EntV antifungal activity. Finally, we show that disulfide bond formation is not a prerequisite for cleavage; EntV cleavage proceeded normally in the absence of DsbA. In conclusion, we present a model in which following secretion, EntV undergoes disulfide bond formation by DsbA and cleavage by GelE in order to generate a peptide capable of inhibiting C. albicans.

## Abstract (precis)

Enterococcus faecalis and Candida albicans are among the most important and problematic pathobionts, organisms that normally are harmless commensals but can cause dangerous infections in immunocompromised hosts. In fact, both organisms are listed by the Centers for Disease Control and Prevention as serious global public health threats stemming from the increased prevalence of antimicrobial resistance. The rise in antifungal resistance is of particular concern considering the small arsenal of currently available therapeutics. EntV is a peptide with antifungal properties, and it, or a similar compound, could be developed into a therapeutic alternative, either alone or in combination with existing agents. However, to do so requires understanding what properties of EntV are necessary for its antifungal activity. In this work, we studied the posttranslational processing of EntV and what modifications are necessary for inhibition of C. albicans in order to fill this gap in knowledge.

## INTRODUCTION

The polymorphic fungus Candida albicans and the Gram-positive bacterium Enterococcus faecalis are opportunistic pathogens capable of causing a variety of infections in susceptible individuals. Both organisms inhabit similar, anatomic niches throughout the human body, such as the gastrointestinal, urogenital, and oral tracts; commonly induce sepsis in hospitalized patients (1); and are often coisolated during infection (2). Importantly, fluconazole-resistant C. albicans and vancomycin-resistant enterococci (VRE) are listed by the CDC as serious public health threats stemming from the increased prevalence of resistance to antimicrobial therapeutics (3).

C. albicans possesses a number of virulence attributes that facilitate pathogenesis. Among them, the ability to form biofilms on biotic surfaces such as the oral cavity and urogenital tract and on abiotic surfaces such as catheters, dental materials, and other medical implants poses significant challenges (4–6). C. albicans biofilms are characterized by dense structures consisting of yeast and hyphal cells that have been encapsulated by a carbohydrate extracellular matrix, thereby preventing optimal access to underlying structures by the immune system and antifungals (5). Thus, new therapies that are effective against biofilms and that could be employed synergistically or in combination with current therapies are needed.

Over the past decade, fungus-bacterium interkingdom associations have become increasingly appreciated for their synergistic and antagonistic effects (7–9). For instance, Pseudomonas aeruginosa phenazines have been shown to kill C. albicans hyphal cells, whereas homoserine lactone quorum-sensing molecules promote the transition of hyphae into the resistant yeast form (10, 11). Conversely, C. albicans has been shown to interact synergistically with the Gram-positive pathogen Staphylococcus aureus or Streptococcus gordonii by promoting increased biofilm biomass (12, 13). In previous work, we reported an antagonistic interaction between C. albicans and E. faecalis using a Caenorhabditis elegans model in which coinfection with both organisms was less virulent than with either organism alone and in a biofilm model in which a factor secreted from E. faecalis inhibited hyphal transformation (14). We identified the factor that antagonizes C. albicans as the Fsr-regulated bacteriocin known as EntV (encoded by *ef1097*) and showed that it protected C. elegans from C. albicans infection. Moreover, EntV significantly reduced inflammation and fungal burden in a mouse model of Oral Pharyngeal Candidiasis (OPC), a biofilm-related infection caused by *Candida* that is commonly observed in immunocompromised individuals (15).

**FIG 1:**
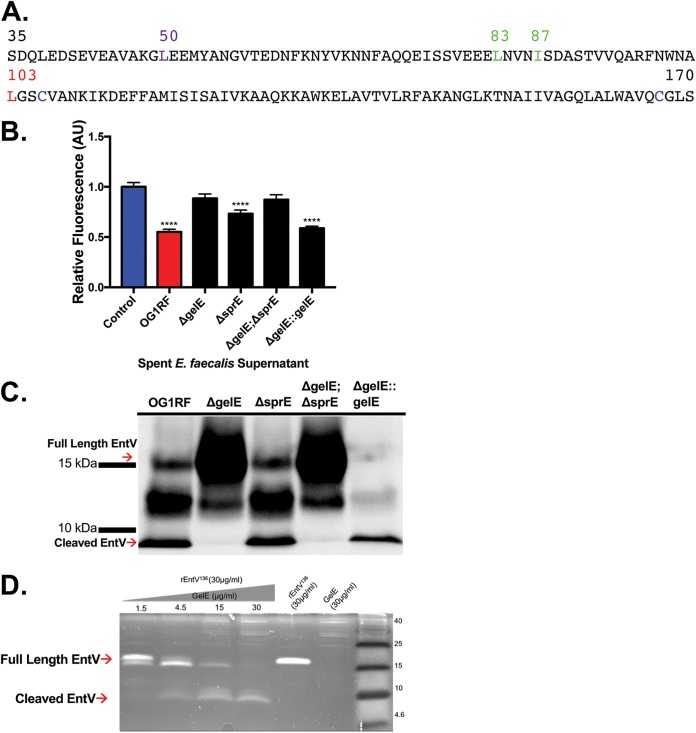
GelE is necessary for inhibition of C. albicans biofilm formation and posttranslational cleavage of EntV. (A) Sequence of secreted EntV (amino acids 35 to 170). Red, first amino acid of the final, cleaved, active form; blue, cysteines that form a disulfide bond; green, intermediate cleaved forms detected by mass spectrometry (*gelE* dependent); purple, intermediate cleaved forms detected by mass spectrometry (*gelE* independent). (B) C. albicans biofilms were exposed to spent E. faecalis supernatants plus artificial saliva (1:1) for 24 h. The OD was measured at 600 nm, and the fluorescence was measured at 488 nm and 530 nm. The data were averaged from 5 independent experiments. **** indicates a *P* value of *<*0.0001 by Student’s two-tailed *t* test compared to the medium control. (C) Western blotting using an anti-EntV antibody was performed on E. faecalis supernatants following precipitation, SDS-PAGE, and membrane transfer. (D) rEntV^136^ (30 μg/ml) was incubated with increasing concentrations of purified GelE (1.5, 4.5, 15, and 30 μg/ml) in PBS at 37°C for 1 h. Samples were subjected to Tricine-SDS-PAGE, stained with SYPRO ruby protein gel stain, and visualized using a 300-nm UV transilluminator.

While the *entV* open reading frame encodes a 170-amino-acid prepropeptide, it was previously reported that the C-terminal 68 amino acids comprise the active form of EntV, required for both bacteriocin (16) and antifungal (15) activities. Although it is assumed that the signal sequence is cleaved during secretion to generate a 136-amino-acid propeptide (Fig. 1A), the downstream processing events that generate the mature peptide are not completely understood. Two proteases regulated by the Fsr system, gelatinase (GelE) and serine protease (SprE), were implicated as being involved using genetic approaches, but whether one or both directly act on EntV has not been determined (14, 16). Additionally, a disulfide bond, which essentially joins the N and C termini of the cleaved form of EntV, was shown to form and be necessary for bacteriocin activity (17), but whether this posttranslational modification is necessary for antifungal activity has not been examined. Finally, how the disulfide bond forms and whether a protein catalyst is involved were unknown.

In this work, we more closely explore the mechanisms by which these posttranslational processing events occur and how they impact EntV’s antifungal activity. We demonstrate that E. faecalis mutants lacking gelatinase activity, but not serine protease activity, fail to generate the 68-amino-acid form of EntV. Additionally, we show that purified GelE can cleave recombinant, full-length (136-amino-acid) EntV *in vitro*, indicating a direct role for this enzyme in EntV cleavage. Moreover, we show that the cysteines within EntV, and the disulfide bond formed between them, are required for antifungal activity. Finally, we provide evidence for the contribution of a previously unstudied, predicted thioredoxin, DsbA, in catalyzing the formation of the disulfide bond within EntV. In conclusion, we provide a characterization of the posttranslational modifications necessary for EntV antifungal activity and the mechanisms by which they occur.

## RESULTS

### GelE cleavage of EntV is necessary for inhibition of C. albicans biofilms.

The secreted and cleaved forms of EntV are shown in Fig. 1A. To investigate the role of cleavage events in EntV antifungal activity, mutants lacking the extracellular proteases gelatinase (GelE) and serine protease (SprE) were tested using isogenic E. faecalis OG1RF mutants and a C. albicans biofilm assay (Fig. 1B). C. albicans biofilms that were incubated with the spent supernatant from either the gelatinase-deficient Δ*gelE* strain or the gelatinase and serine protease double mutant Δ*gelE* Δ*sprE* strain yielded robust biofilms that were identical to the control (medium-only) biofilm. Conversely, those C. albicans biofilms that were incubated with the complemented Δ*gelE*::*gelE* strain suppressed biofilm formation, similar to the wild type (WT) (OG1RF). The spent supernatant from a Δ*sprE* strain also significantly inhibited biofilm formation, but the phenotype was not as strong as those of the strains containing a *gelE* deletion. Overall, the results indicate that GelE plays a major role in generating the fully active EntV peptide, with SprE as a contributing factor.

To monitor the processing of EntV biochemically, trichloroacetic acid (TCA)-precipitated proteins from supernatants were subjected to Western blotting with anti-EntV serum (Fig. 1C). From the OG1RF parental strain, cleaved EntV (7.2 kDa) was detectable. The full-length secreted form of the protein (14.8 kDa) was also visible, although it tended to run above its molecular weight. Multiple intermediate products, corresponding to molecular weights of around 10 to 13 kDa, were also apparent (see below). No bands were detected in supernatants from a Δ*entV* strain (see Fig. 4C). Interestingly, the fully cleaved form of EntV (7.2 kDa) was observed only in those strains that contained a functional GelE: the wild-type, Δ*sprE*, and Δ*gelE*::*gelE* strains. Notably, the pattern of EntV processing in supernatants from the *ΔsprE* strain is identical to that of the WT. The *ΔsprE* deletion strain TX5243 was not able to be complemented. To verify that the phenotype was linked to the mutation and not spurious, we generated a second, independent deletion strain employing a different targeting vector. The strain, TX5431, both behaved identically to TX5243 in the biofilm assay (see Fig. S1A in the supplemental material) and also displayed normal cleavage of EntV (Fig. S1B). The results support a model in which GelE, but not SprE, is required to cleave EntV into the form active against C. albicans biofilm.

10.1128/mBio.01334-19.1 FIG S1 Two independent Δ*sprE* strains (TX5243 and TX5431) show intermediate biofilm phenotypes and unaffected EntV cleavage. (A) C. albicans biofilms were exposed to spent E. faecalis supernatants for 24 h. The data were averaged from 3 independent experiments. **** indicates a *P* value of *<*0.0001 by Student’s two-tailed *t* test compared to the medium control. (B) Hybridization using an anti-EntV antibody was performed on TCA-precipitated supernatants from the indicated strains following precipitation, SDS-PAGE, and membrane transfer. Download FIG S1, TIF file, 2.9 MB. Copyright © 2019 Brown et al. 2019 Brown et al. This content is distributed under the terms of the Creative Commons Attribution 4.0 International license.

What, then, explains the partial effects of the Δ*sprE* mutant in the biofilm assay (Fig. 1B)? Since the *gelE* and *sprE* genes are cotranscribed (18), we postulated that the deletion of *sprE* might reduce the stability of the transcript or cause some other alteration that reduces the levels of the *gelE* message. Indeed, reverse transcription-quantitative PCR (qRT-PCR) with primers specific to *gelE* shows that there are significantly lower levels of the *gelE* transcript in the Δ*sprE* strain than in the wild-type or Δ*gelE* complemented strain (Fig. S2), supporting this hypothesis. However, we do not observe a discernible decrease of the amount of cleaved EntV as might be predicted if there were less GelE (Fig. 1C and Fig. S1B). It might be that the effect of lower levels of GelE are apparent only during earlier growth phases. Alternatively, SprE could affect C. albicans biofilm independently of the EntV cleavage event. In conclusion, while we favor a model in which the apparent effect of SprE loss is due an indirect lowering of GelE levels, we were unable to confirm this postulate in these experiments.

10.1128/mBio.01334-19.2 FIG S2 Lower *gelE* expression levels are observed in the Δ*sprE* mutant than in OG1RF (wild-type) E. faecalis. RT-PCR analysis of *gelE* expression relative to *gyrA* was performed on each strain at the 8- and 16-h time points. Data are representative of results from three independent experiments, with each sample analyzed in triplicate. Student’s two-tailed *t* test was used to compare relative transcript levels between the WT and the indicated strains. ***, *P ≤ *0.0002; **, *P ≤ *0.001. Download FIG S2, TIF file, 2.9 MB. Copyright © 2019 Brown et al. 2019 Brown et al. This content is distributed under the terms of the Creative Commons Attribution 4.0 International license.

Rather, to further verify that GelE directly cleaves EntV, we examined EntV processing in an *in vitro* assay using purified proteins. GelE was purified from E. faecalis (19), whereas the propeptide, the 136-amino-acid form of EntV, was generated recombinantly in Escherichia coli (15). We added increasing concentrations of GelE to 30 μg/ml of purified EntV. After stopping the reactions, the samples were run on a gel and stained (Fig. 1D). Full-length EntV was clearly visible running in the lane lacking GelE. Upon the addition of GelE, dose-dependent cleavage of EntV was observed. At the highest concentration, the 7.2-kDa form of EntV was the most prevalent (Fig. 1D). However, the amount of cleaved GelE was smaller than the amount of full-length starting material, suggesting that GelE might completely digest EntV given enough time. Such a phenomenon was previously observed in a study of GelE cleavage of AtlA (20).

In both the *in vivo* and *in vitro* examinations of EntV cleavage, products of an intermediate size (10 to 13 kDa) were observed (Fig. 1C and D). Some appeared to not be completely dependent on GelE, as bands in this range were still observed, albeit at lower levels, in the Δ*gelE* mutant (Fig. 1C). To characterize these cleavage products and determine which ones were GelE dependent, supernatants from wild-type and Δ*gelE* strains were run on gels, and gel slices corresponding to these intermediates were excised and examined by liquid chromatography-mass spectrometry (LC-MS) analysis. The experiments revealed that once processed during secretion into the propeptide form beginning at Ser^35^, some EntV undergoes a GelE-independent cleavage event at Leu^50^, as this form was detected in both the wild-type and Δ*gelE* samples. However, there appear to be at least two other GelE-dependent cleavage events that can occur at Leu^83^ and Ile^87^, in addition to the cleavage event that occurs at Leu^103^ to generate the active form, as these cleavage products were detected only in the wild-type sample (Fig. 1A).

**FIG 2:**
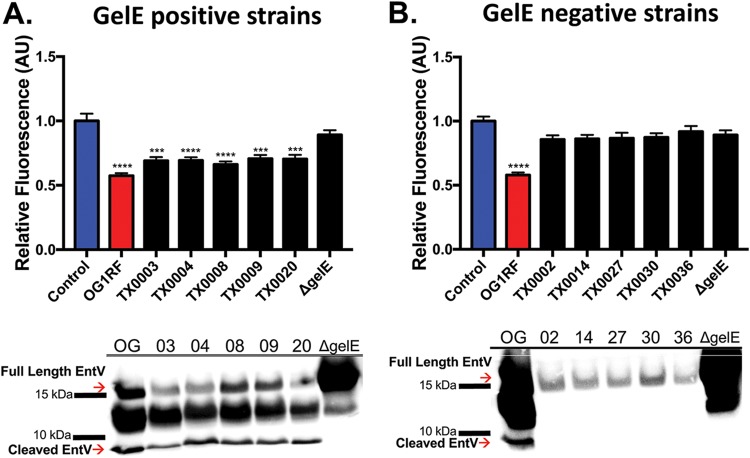
Presence of GelE is correlated with inhibition of C. albicans biofilm and processing of EntV in clinical isolates. C. albicans biofilms were exposed to E. faecalis supernatants of previously reported strains positive (A) and negative (B) for GelE activity for 24 h. The data were averaged from 5 independent experiments. **** indicates a *P* value of *<*0.0001 by Student’s two-tailed *t* test compared to the Δ*gelE* strain. Western blotting using an anti-EntV antibody was performed on E. faecalis supernatants following precipitation, SDS-PAGE, and membrane transfer.

Many isolates of E. faecalis are gelatinase negative, as determined by their ability to digest gelatin added to agar plates (21). We reasoned that these strains could be used to further investigate the role of GelE in the processing of EntV into the biofilm-inhibiting form. Indeed, all E. faecalis clinical isolates with confirmed GelE activity by gelatinase digestion significantly suppressed C. albicans biofilm formation. Additionally, significant levels of cleaved EntV were detectable by Western blot analysis (Fig. 2A). However, clinical isolates lacking GelE activity were attenuated in their ability to suppress C. albicans biofilm formation and lacked the ability to fully cleave EntV (Fig. 2B). Thus, the requirement for GelE and EntV cleavage appears to be a common feature for suppression of C. albicans biofilm formation by E. faecalis.

### Disulfide bond formation in EntV is necessary for inhibition of C. albicans.

**FIG 3:**
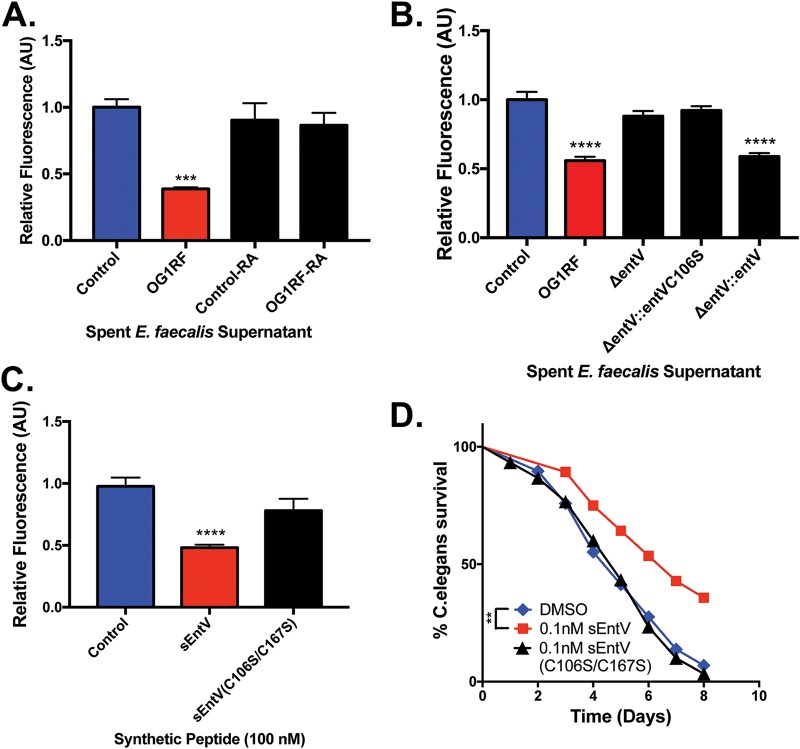
Disrupted disulfide bond formation in EntV results in loss of activity against C. albicans biofilms and infection. (A to C) C. albicans biofilms were exposed to the spent wild-type E. faecalis supernatant or the spent supernatant that was reduced/alkylated (RA) (A), spent E. faecalis supernatants from the mutants indicated (B), or artificial saliva medium containing a synthetic EntV peptide in the native form or with disrupted disulfide bonding (C). Asterisks indicate a *P* value of *<*0.0001 by Student’s two-tailed *t* test compared to the medium control. For spent-supernatant experiments, the data were averaged from 4 independent experiments, while 5 independent experiments were used for biofilms treated with the synthetic peptide. (D) C. elegans was exposed to C. albicans on solid medium and then exposed to wild-type EntV or the C106S mutant peptide in liquid medium. Survival was scored daily. Significant protection compared to the vehicle control was observed with the wild type at concentrations of 0.1 and 0.01 nM. **, *P < *0.01.

EntV contains two cysteines (Cys^106^ and Cys^167^) that form a disulfide bond. In previous work, differential S-alkylation in the presence or absence of a reducing agent demonstrated that EntV contained a disulfide bond that was essential for bacteriocin activity (17). To determine if this disulfide bond is also important for antifungal activity, we tested the ability of the reduced and alkylated spent supernatant from OG1RF to suppress C. albicans biofilm formation. As shown in Fig. 3A, E. faecalis supernatants treated in such a manner no longer inhibited C. albicans biofilm formation, consistent with disulfide bond formation being necessary for EntV antifungal activity. In a more targeted approach, we complemented a Δ*entV* mutant with a mutated version of the gene to generate the Δ*entV*::*entVC106S* strain. The spent supernatants from the Δ*entV* and Δ*entV*::*entVC106S* strains were unable to suppress C. albicans biofilm formation relative to the wild type and complemented (Δ*entV*::*entV*) E. faecalis strains (Fig. 3B).

In previous work, we synthesized the EntV peptide, including the disulfide bond, and showed that it inhibited C. albicans biofilm formation and hyphal morphogenesis and was protective against C. albicans in animal models of infection (15). To test the importance of this bond, a synthetic version of EntV lacking both cysteines (C106S and C167S) was generated. As shown in Fig. 3C, this form of the peptide had no activity against C. albicans biofilm formation (Fig. 3C). Next, we performed a Caenorhabditis elegans survival assay by treating nematodes infected with C. albicans with either the wild-type or the C106S/C167S synthetic EntV peptide. As observed previously, the animals treated with the wild-type form of the EntV peptide lived significantly longer than those exposed to the vehicle control, but those treated with EntV(C106S/C167S) were not protected (Fig. 3D). In total, these results demonstrate that the EntV disulfide bond is critical for activity against C. albicans.

### The disulfide-bond-forming protein DsbA is necessary for EntV activity.

**FIG 4:**
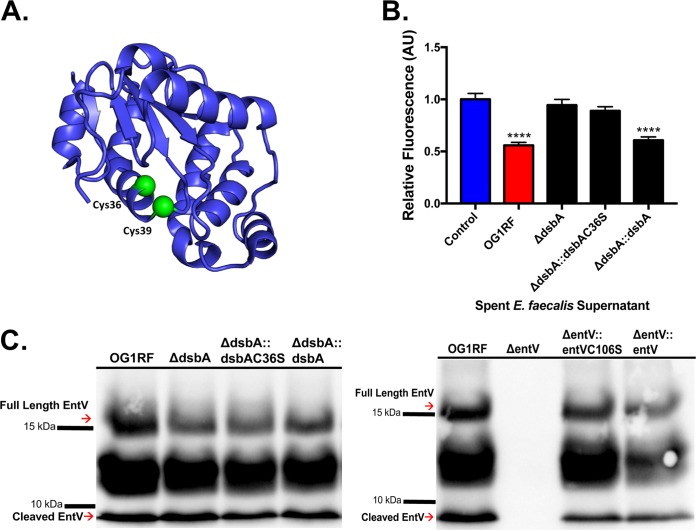
DsbA is required for EntV activity. (A) PyMOL image of E. faecalis DsbA (V583) with cysteines highlighted (PDB accession number 1Z6M). (B) C. albicans biofilms were exposed to spent E. faecalis supernatants from the indicated strains for 24 h. The data were averaged from 4 independent experiments. **** indicates a *P* value of *<*0.0001 by Student’s two-tailed *t* test compared to the medium control. (C) Hybridization using an anti-EntV antibody was performed on E. faecalis supernatants from the indicated strains following precipitation, SDS-PAGE, and membrane transfer.

Having established that disulfide bond formation within EntV is critical for antifungal activity, we next asked if a specific protein catalyzes the reaction, thereby contributing to the maturation of the active peptide. In Gram-negative bacteria, disulfide bonds in periplasmic proteins are formed via catalysis by thioredoxins of the DsbA family. Extracellular disulfide formation is less explored and thought not to be as biologically important in *Firmicutes* such as E. faecalis (22). *In silico* analysis of the OG1RF genome revealed the presence of a gene encoding a thioredoxin of the DsbA family of proteins. In fact, the structure of the protein was previously solved by X-ray crystallography by a consortium and has a fold very consistent with this function, comprising a canonical thioredoxin domain with the conserved CXXC active-site cysteines arranged with glutaredoxin topology (23) (Fig. 4A). However, the gene that encodes this protein has never been functionally studied in E. faecalis, and no specific roles have been described.

Therefore, to examine the contribution of the E. faecalis thioredoxin to the posttranslational modification of EntV, we generated a Δ*dsbA* mutant, a complemented strain *(*Δ*dsbA*::*dsbA*), and a strain complemented with a C36S mutation (Δ*dsbA*::*dsbAC36S*) predicted to abrogate one of the active-site cysteine residues. The strains were tested in the C. albicans biofilm assay. C. albicans biofilms that were treated with the spent supernatant from either the Δ*dsbA* or Δ*dsbA*::*dsbAC36S* strain grew unabated to control levels, while C. albicans biofilm suppression was observed when biofilms were treated with supernatants from either the wild-type or complemented (Δ*dsbA*::*dsbA*) strain (Fig. 4B). Thus, the data suggest that DsbA and its oxidoreductase activity are critical for EntV activity.

An interesting question posed by these findings is whether disulfide bond formation is required for cleavage by GelE, as the disulfide bond forms only 3 amino acids away from where the peptide is cleaved (Fig. 1A). Therefore, we examined EntV cleavage in the Δ*dsbA* and Δ*entV* mutant strains. All strains (with the exception of the Δ*entV* strain) were fully cleaved irrespective of their ability to form disulfide bonds in EntV, as the Δ*entV*::*entVC106S* and *ΔdsbA* strains still displayed normal cleavage (Fig. 4C). Taken together, these data indicate that while DsbA is critical for generating a disulfide bond necessary for active EntV, cleavage of EntV occurs in a DsbA-independent manner.

## DISCUSSION

**FIG 5:**
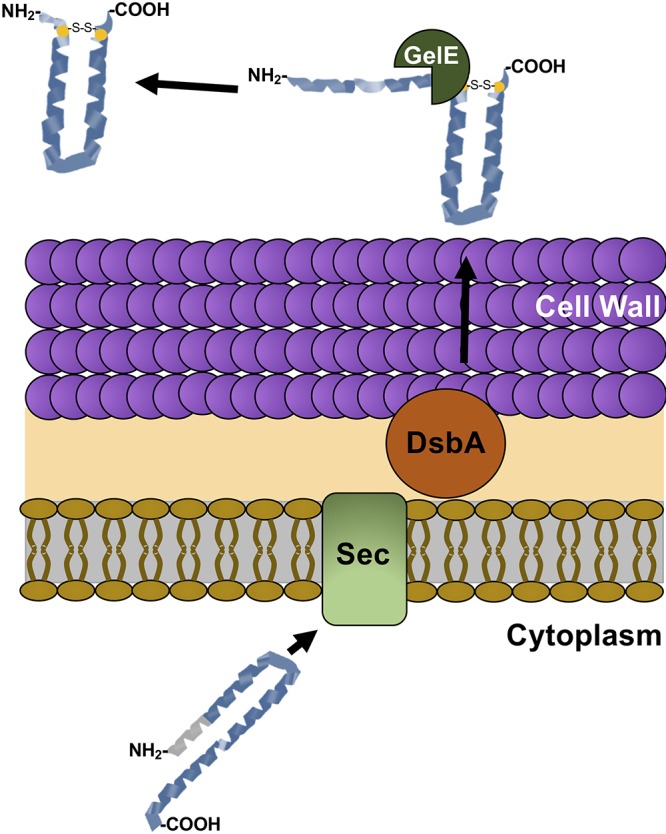
Model of EntV processing. Following translation, the signal peptide (gray) directs EntV to the Sec system for secretion and signal peptide removal. DsbA, predicted to be associated with the cell envelope, forms the disulfide bond between the two redox-active cysteines (yellow). Following secretion, the extracellular protease GelE removes the rest of the N terminus, generating the 68-amino-acid, active form of the protein.

Hyphal differentiation is crucial for biofilm formation and C. albicans pathogenesis (24–26). In previous work, we showed that the bacteriocin EntV is secreted by E. faecalis and is capable of suppressing C. albicans hyphal morphogenesis (15). Here we extend those observations by detailing some of the posttranslational modification events necessary for cleavage and full activity of EntV. A model for how EntV is processed is presented in Fig. 5. Briefly, nascent EntV contains a signal peptide that is predicted to direct its secretion across the Sec translocase, where it interacts with DsbA associated with the cell envelope, as previously shown in other bacteria (22, 27). Once the peptide is secreted, gelatinase, whose nascent form also contains a signal peptide and has been characterized as an extracellular protease (28, 29), cleaves EntV from its full-length size of 14.8 kDa to the 7.2-kDa form that suppresses C. albicans hyphal morphogenesis by an unknown mechanism.

Gelatinase is an important virulence factor that was shown to contribute to virulence in most animal models in which it has been tested, including C. elegans, insects, and a variety of vertebrate animal infection models (30). GelE is a member of the M4 family of proteases and is similar to thermolysin from Bacillus thermoproteolyticus and aureolysin from S. aureus (31, 32). With broad substrate specificity, GelE is known to cleave gelatin; enterococcal proteins, including the conjugative sex pheromones; and host factors such as insulin β-chain, endothelin, hemoglobin, fibrinogen, fibronectin, collagen, and laminin (29, 33). Furthermore, GelE interferes with the innate immune response by inactivating the human antimicrobial peptides LL-37 and β-defensins and is pertinent for E. faecalis dissemination (21, 34, 35). Moreover, a role for GelE has been observed in biofilm development and regulation of autolysis in E. faecalis (36). To this list of roles, we can now add that GelE directly processes EntV into its active, antifungal form. While our work in the C. elegans model (14) and our biofilm assays here suggested that the serine protease SprE might also be involved, we attribute the effects of an *sprE* mutant in these assays to possibly be the result of an indirect effect on *gelE* expression (see Fig. S2 in the supplemental material). *In vitro* assays demonstrating that GelE directly cleaves EntV (Fig. 1D) and the fact that strains that do not generate GelE also do not process EntV (Fig. 2) provide strong evidence that GelE is the primary protease that cleaves EntV.

Despite the fact that gelatinase is a factor that appears to benefit E. faecalis in the host environment, approximately 30 to 70% of E. faecalis strains isolated from humans do not produce gelatinase (37). Could the presence or absence of gelatinase be selected for because of its role in EntV processing and, by extension, effects on interactions with other microbes? Cleavage of EntV by gelatinase is necessary for its bacteriocin activity (16), and here we demonstrated that it is necessary for its role in modulating C. albicans hypha formation. Interestingly, the gut microbiomes of antibiotic-treated mice that are subsequently gavaged with C. albicans become dominated by E. faecalis. However, the C. albicans-induced gastritis in gnotobiotic mice, which do not experience this overgrowth of E. faecalis, is much worse (38). These data suggest that E. faecalis may promote a commensal association with C. albicans in the mammalian gut, and it would be interesting to ask if this association is GelE dependent. In contrast, the coisolation of C. albicans and E. faecalis in human infection is common (39–41). For example, an extensive study examining patients with candidemia found that 24% had synchronous bacteremia, of whom 27% were infected with *Enterococcus* spp., a dramatic number considering that the second most prevalent species was E. coli, at 7% (40). Based on these data, it would be interesting to study if the presence of gelatinase, and, by extension, processed EntV, is less prevalent in strains of E. faecalis found associated with C. albicans infections.

In addition to GelE, we also demonstrated that DsbA is necessary for EntV antifungal activity. While DsbA has been known to exist within the genome of E. faecalis for over a decade (23, 42, 43), this is first report to show its contribution to the posttranslational modification of a protein. While Gram-positive organisms like E. faecalis do not have a periplasm where oxidative folding can occur, the region between the cell membrane and the cell wall has been postulated to serve a similar purpose, and DsbA-like proteins have been localized to the cell membrane fraction (22, 27). Therefore, as proteins like EntV are secreted, DsbA is likely positioned to catalyze the requisite disulfide bonds needed for activity. However, disulfide bond formation in *Firmicutes* like E. faecalis is thought not to be a biological imperative, in contrast to the *Actinobacteria* and the majority of Gram-negative organisms (22). Species of *Staphylococcus*, *Lactococcus*, and *Streptococcus* secrete few proteins containing cysteines (44, 45). Some species, like Streptococcus pneumoniae, do not encode any thiol-disulfide oxidoreductases (44, 45). A deletion of the only DsbA-like protein in S. aureus resulted in no obvious phenotype, although further careful study revealed that it is necessary for proper surface display of the pseudopilin ComGC (27, 46). Interestingly, and relevant to this work, thiol-disulfide oxidoreductases have been associated with specific bacteriocins in *Firmicutes*. Bacillus subtilis encodes the putative oxidoreductases BdbA and BdbB in the same operon that encodes sublancin 168, a bacteriocin with disulfide bonds (47). Some strains of E. faecalis (not OG1RF) contain a gene for a bacteriocin called F4-9 that resides adjacent to a gene postulated to encode a thiol-disulfide oxidoreductase necessary for its proper folding (48). In contrast, *entV* and *dsbA* are part of the core E. faecalis genome and are not near each other on the chromosome, suggesting that DsbA has a larger biological role in E. faecalis and is important for the oxidative folding of proteins other than EntV.

In conclusion, we demonstrated that cleavage by the protease GelE and disulfide bond formation by the thiol-oxidoreductase DsbA are necessary for EntV activity against C. albicans biofilm formation. Since fungal infections resistant to current therapeutics are becoming increasingly problematic, particularly with biofilms that form on implanted medical devices, it is important to identify new compounds with different mechanisms of action. By understanding the posttranslational processing of EntV and what features are important for its activity, we will be better equipped to develop EntV, or a similar compound, into a therapeutic. In future studies, we hope to elucidate the structure of EntV at high resolution and determine the mechanism of action that it employs to inhibit C. albicans hypha formation.

## MATERIALS AND METHODS

### Bacterial strains.

All strains, plasmids, and oligonucleotides are listed in Table S1 in the supplemental material (49–53). The Δ*dsbA* mutant was constructed as previously described to create an in-frame markerless deletion using a P-pheS* counterselection system (54). Briefly, sequences flanking the 5′ and 3′ ends of *dsbA* were amplified by PCR. The 2 products were fused together by PCR with primer-introduced BamHI and NotI sites. The resulting fragment was ligated into a BamHI/NotI-digested pCJK47 vector, resulting in pAOBD1. pAOBD1was electroporated into CK111 cells, CK111 containing pAOBD1 was then mated to OG1RF, and counterselection was completed to create the OG1RF Δ*dsbA* strain. Deletion of *dsbA* was confirmed by PCR and sequencing. The *dsbA*-complemented strain (Δ*dsbA*::*dsbA*) was constructed by first amplifying the wild-type *dsbA* locus and incorporating BamHI and NotI sites. The fragment was digested and ligated into a BamHI- and NotI-digested pKK6 plasmid, creating pAOBD2. pAOBD2 was electroporated into CK111 cells and then mated to the Δ*dsbA* strain to create the Δ*dsbA*::*dsbA* strain. Complementation of *dsbA* was confirmed by PCR and sequencing. In a similar fashion, the Δ*dsbA*::*dsbAC36S* and Δ*entV*::*entVC106S* mutants were created by site-directed mutagenesis in Cys^36^ of *dsbA* or in Cys^106^ of *entV* using the previously generated pAOBD2 plasmid containing wild-type *dsbA* or pCEG1 containing entV* with a Silence mutation to generate AOBD36 and AOBCG1, respectively (15). Once the respective plasmids containing cysteine-to-serine mutations were created, they were electroporated into CK111 cells and then mated to a strain in either the Δ*dsbA* or Δ*entV* background to generate the Δ*dsbA*::*dsbAC36S* and Δ*entV*::*entVC106S* mutants. Both mutants were confirmed by PCR and sequencing. A second *gelE* disruption mutant (TEX5431) was generated by cloning an intragenic region of *gelE*, different from that used to make TX5243, into a targeting vector according to a previously described procedure to obtain mutant strain TX5431 (18). The clinical isolates were previously described (21).

10.1128/mBio.01334-19.3 TABLE S1 Strains, plasmids, and oligonucleotides used in this study. Download Table S1, DOCX file, 0.03 MB. Copyright © 2019 Brown et al. 2019 Brown et al. This content is distributed under the terms of the Creative Commons Attribution 4.0 International license.

### Mass spectrometry analysis.

Trichloroacetic acid-precipitated proteins from the spent supernatant of E. faecalis were subjected to SDS-PAGE. Bands corresponding to the 11- to 13-kDa size range were excised and sent to The University of Texas Health Science Center Clinical and Translational Proteomics Service Center for in-gel spot analysis by liquid chromatography-mass spectrometry (LC-MS) (for sample preparation and analysis, see below). Cleavages of EntV peptides were compared between EntV samples obtained from the WT (GelE^+^) and a *gelE* mutant (GelE^−^), as a control. Only peptide cleavage events that were unique to the WT samples and that had cleavage at sites not corresponding to trypsin cleavage were accepted as true cleavage events.

### Sample preparation for LC-MS/MS, data processing, and analysis.

The gel band samples were subjected to in-gel digestion (55). An aliquot of the tryptic digest (in 2% acetonitrile–0.1% formic acid in water) was analyzed by LC-tandem MS (MS/MS) on an Orbitrap Fusion Tribrid mass spectrometer (Thermo Fisher Scientific) interfaced with a Dionex UltiMate 3000 binary Rapid Separation Liquid Chromatography (RSLC) nano system. Peptides were separated onto an Acclaim PepMap C_18_ column (0.075-mm by 150-mm internal diameter; 3 μm) at a flow rate of 300 nl/min. Gradient conditions were 3% to 22% solvent B for 40 min, 22% to 35% solvent B for 10 min, 35% to 90% solvent B for 10 min and 90% solvent B held for 10 min (solvent A, 0.1% formic acid in water; solvent B, 0.1% formic acid in acetonitrile). The peptides were analyzed using a data-dependent acquisition method, and the Orbitrap Fusion instrument was operated with measurement of Fourier Transform Mass Spectroscopy-1 (FTMS1) at resolutions of 120,000 full width at half maximum (FWHM), a scan range of *m/z* 350 to 1,500, AGC target 2E5, and a maximum injection time of 50 ms. During a maximum 3-s cycle time, the Ion Trap Mass Spectrocopy (ITMS)-2 spectra were collected in the rapid-scan-rate mode, with Collision-induced dissociation (CID) Normalized-collision energy (NCE) 35, a 1.6 *m/z* isolation window, automatic gain control target 1E4, and a maximum injection time of 35 ms, and dynamic exclusion was employed for 35 s. The raw data files were processed using Thermo Scientific Proteome Discoverer software version 1.4, and spectra were searched against the Enterococcus faecalis database using the Sequest search engine with no enzyme selection. Search results were trimmed to a 1% false discovery rate (FDR) for strict and a 5% FDR for relaxed conditions using Percolator. MS tolerance was set 10 ppm, and MS/MS tolerance was set to 0.8 Da. Carbamidomethylation on cysteine residues was used as a fixed modification, and oxidation of methionine as well as phosphorylation of serine, threonine, and tyrosine were set as variable modifications.

### *In vitro* biofilm assay.

Biofilm assays were performed as described in our previous report, using standard techniques (15). Briefly, C. albicans (HWP1p::GFP hypha-specific reporter strain) biofilms were first grown in a black clear-bottom Costar 96-well plate for 1.5 h at 30°C in 1× phosphate-buffered saline (PBS) and then treated with the spent E. faecalis M9HY supernatant for 24 h in artificial saliva medium (at a 1:1 ratio of artificial saliva medium to spent M9HY medium). After 24 h, the medium was removed, and the wells were washed once with 1× PBS and then resuspended in 100 μl of 1× PBS. In synthetic peptide experiments, artificial saliva medium was supplemented with 100 nM synthetic peptide. The relative fluorescence was calculated by first measuring the absorbance of each well at 600 nm, followed by measuring the fluorescence at an excitation wavelength of 488 nm and an emission wavelength of 530 nm. Biofilm values were calculated by determining the ratio of fluorescence (488 nm/530 nm) to absorbance (optical density at 600 nm [OD_600_]). This fluorescence ratio was then normalized to the value for the M9HY non-spent-supernatant control. Each experiment was conducted in a 96-well plate, using 4 replicates per strain. All experiments were repeated on four separate occasions, and values from all experiments were averaged and used for statistical analysis. The different treatment types per experiment were compared by pairwise Student’s *t* test or one-way analysis of variance (ANOVA), with a *P* value of <0.05 considered statistically significant, using GraphPad Prism (version 7.0).

### Nematode infection and synthetic EntV treatment model.

C. elegans *glp-4*(bn2); *sek-1*(km4) nematodes were propagated by using standard techniques on E. coli strain OP50 on nematode growth medium (NGM) agar as described in previous reports (15, 56). The liquid infection assay was performed as described previously (14, 57). Briefly, young adults were incubated for 4 h on solid medium with the wild-type C. albicans SC5314 strain, and the worms were then collected, washed, and transferred to 6-well plates containing 20% brain heart infusion (BHI) broth and 80% M9 Buffer for Worms (M9W) with various concentrations of EntV peptide, after which viability was assayed daily.

### Precipitation of proteins from the supernatant and visualization by Western blotting.

E. faecalis cultures were grown for 16 h at 37°C. The spent supernatant was then treated with 5% TCA for 48 h at 4°C. Precipitated proteins were centrifuged at 10,000 rpm for 10 min, and the supernatant was then discarded. This step was repeated twice in order to ensure that all TCA was removed prior to solubilization in 6 M urea. TCA-precipitated proteins were separated by SDS-PAGE, and Western blotting was performed using a rabbit polyclonal anti-EntV antibody at a concentration of 1:1,000 and a horseradish peroxidase (HRP)-conjugated goat anti-rabbit antibody at a concentration of 1:10,000. The Western blots were processed using Thermo Fisher Scientific SuperSignal West Dura extended-duration substrate and imaged using the GE ImageQuant LAS4000 mini imaging system. To generate anti-EntV serum, full-length protein was recombinantly expressed from strain CEGE1 (15). Cell lysates were run on an SDS-PAGE gel, and the overexpressed protein was excised and sent to Cocalico Biologicals, Inc., for antibody generation.

### Proteolytic cleavage of rEntV136 by GelE.

GelE was purified as previously described by Pinkston et al. (19). Recombinant EntV136 (rEntV136) (30 μg/ml) was incubated with increasing concentrations of purified GelE (1.5, 4.5, 15, and 30 μg/ml) in PBS at 37°C for 1 h. Samples were boiled at 100°C for 5 min in SDS-PAGE sample buffer with β-mercaptoethanol (BME) to terminate the reaction. To evaluate cleavage of rEntV136, reactions were subjected to Tricine-SDS-PAGE, and samples were stained with SYPRO ruby (Lonza) protein gel stain and visualized using a 300-nm UV transilluminator (58).

### Reduction and alkylation of the supernatant.

The spent supernatant was first reduced by the addition of 0.2% 2-mercaptoethanol (Millipore-Sigma) and incubated at room temperature for 1 h. Next, 0.2% 4-vinylpyridine (Millipore-Sigma) was added, and incubation continued at room temperature for an additional 2 h. One milliliter of medium was then added to 3-kDa Nominal Molecular Weight Limit (NMWL) Amicon Ultra tubes (Millipore-Sigma) and spun at 14,000 × *g* for 30 min. The remaining concentrate was washed three times with 0.4 ml of 5 mM HEPES buffer and resuspended in 0.5 ml of HEPES. The reduced and alkylated medium was then added to artificial saliva at a 1:1 ratio (15).
