# Supplementary figures and images for: Antifungal Activity of the Enterococcus faecalis Peptide EntV Requires Protease Cleavage and Disulfide Bond Formation
Source: mBio. 2019 Jul 2;10(4):e01334-19. doi: 10.1128/mBio.01334-19 (PMC6606811; doi:10.1128/mBio.01334-19)

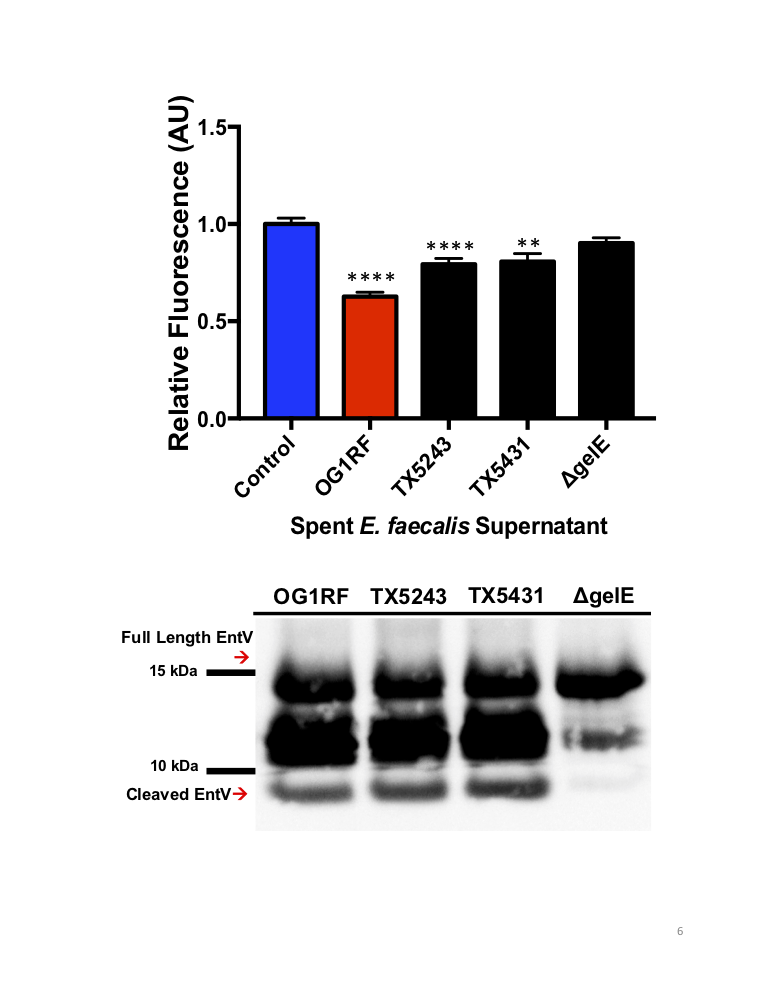

Supplement: FIG S1 [file mBio.01334-19-sf001.tif]

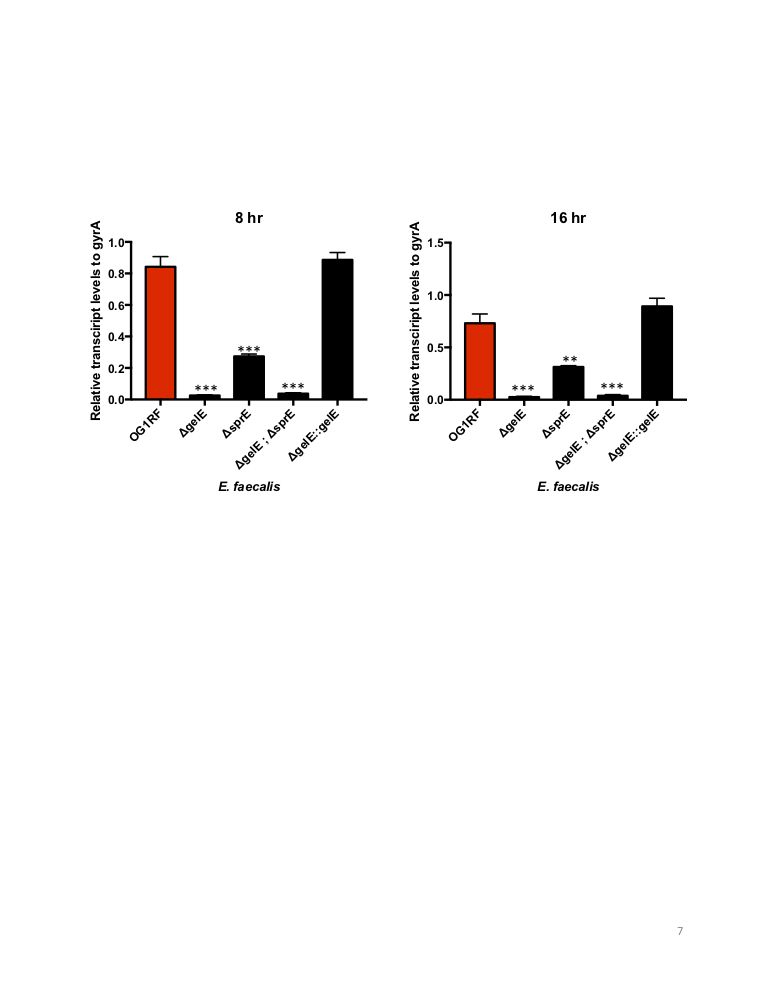

Supplement: FIG S2 [file mBio.01334-19-sf002.tif]
