# Supplementary material for: Antifungal Activity of the Enterococcus faecalis Peptide EntV Requires Protease Cleavage and Disulfide Bond Formation
Source: mBio. 2019 Jul 2;10(4):e01334-19. doi: 10.1128/mBio.01334-19 (PMC6606811; doi:10.1128/mBio.01334-19)
Supplement: TABLE S1 [file mBio.01334-19-st001.docx]

**Table S1. Strains, plasmids and oligos used in this study**

|  |  |  |
| --- | --- | --- |
| \| *C. albicans*  strain \| HWP1-GFP \| ura3/ura3ENO1/eno1::HWP1p-GFP-URA3 \| SC5314 \| [49] \| \| --- \| --- \| --- \| --- \| --- \| \| ***Enterococcus faecalis* Strains** \| \| \| \| \| \| **Strain** \| **Genotype** \| **Characteristics** \| **Parent** \| **Source** \| \| OG1RF \| wild-type \| Gel+ Spr+ Rf^R^ Fa^R^ \| OG1 \| [50] \| \| TX5264 \| $\Delta$*gelE* \| Gel- Spr+ Rf^R^ Fa^R^ \| OG1 \| [51] \| \| TX5439 \| $\Delta$*gelE::gelE* \| Gel+ Spr+ Rf^R^ Fa^R^ Ery^R^ \| OG1 \| [21] \| \| TX5243 \| $\Delta$*sprE* \| Gel+ Spr- Rf^R^ Fa^R^ Kan^R^ \| OG1 \| [18] \| \| TX5431 \| $\Delta$*sprE* \| Gel+ Spr- Rf^R^ Fa^R^ Kan^R^ \| OG1 \| This Study \| \| TX5128 \| $\Delta$*gelE*$;\Delta$*sprE* \| Gel- Spr- Rf^R^ Fa^R^ Kan^R^ \| OG1 \| [18, 52] \| \| AOBD1 \| $\Delta$*dsbA* \| Gel+ Spr+ Rf^R^ Fa^R^ \| OG1 \| This Study \| \| AOBD2 \| $\Delta$*dsbA::dsbA* \| Gel+ Spr+ Rf^R^ Fa^R^ \| OG1 \| This Study \| \| AOBD36 \| $\Delta$*dsbA::dsbAC36S* \| Gel+ Spr+ Rf^R^ Fa^R^ \| OG1 \| This Study \| \| AOBCG1 \| $\Delta$*entV::entVC106S* \| Reconstituted *entV* containing a C106S mutation into a plasmid containing *entV* with a silent nucleotide change*, $\Delta$entV [*entV*^*^], Rf^R^ Fa^R^ \| OG1 \| [15] and This Study \| \| **Plasmids** \| \| \| \| \| \| pKK6 \| PCR products containing *dsbA* (519 bp), *dsbA*::C36S (519 bp), *entV*::C106S (513) and its promoter were cloned into the vector. \| \| \| [53] \| \| pCJK47 \| PCR product containing flanking sequences of *dsbA* (594 bp upstream, and 687 bp downstream) were cloned into the vector for Gibson Assembly. \| \| \| [54] \| \| pTEX4577 \| PCR product containing 690 bp *sprE* fragment was cloned into pAT18 shuttle vector to create this vector used to generate sprE disruption in TX5431 \| \| \| This study \|   **Oligos (Primers)** | | |
| **Primer Name** | **Sequence** | **Primer use** |
| dsbA_Comp_Forward | CAAATAAAAAGCGGCCGCATGGATATTTCAGTAATTGATGCAACAAAAGTTAACAC | *dsbA* complement |
| dsbA_Comp_Reverse | CGACGTCATATGGATCCTTATTTTTCAATGTATCCTCGTAATTCTTCTTCAGTGAC | *dsbA* complement |
| Frag1_Forward_dsbA | AGAATCGCTAGTTCTAGAGCGGCCGCGGGCAATATACTGCGGAAACATGT | Gibson assembly *dsbA* deletion mutant |
| Frag1_Reverse_dsbA | TTCCATTTTTAAAAAAACCTCATCCTTTCAAACCTTTATTTCTCTTTTAGTATACCG | Gibson assembly *dsbA* deletion mutant |
| Frag2_Forward_dsbA | AGGTTTTTTTAAAAATGGAAACAGCGAGTGTCG | Gibson assembly *dsbA* deletion mutant |
| Frag2_Reverse_dsbA | AGACGCGTCGACGTCATATGGATCCTAGTATAACATAATCCAAAAGTTTCGGATAAAAAATGACACAAAA | Gibson assembly *dsbA* deletion mutant |
| C36S_Forward_dsbA | aatttatcaatgttcgctccccttattgcagaaaatggtttgaag | C36S *dsbA* mutant |
| C36S Reverse_dsbA | taaggggagcgaacattgataaattctatcattttgacaggggcattg | C36S *dsbA* mutant |
| C106S_Forward_entV | attaggaagttccgttgctaacaaaatcaaagatgagttttttg | C106S *entV* mutant |
| C106S_Reverse_entV | gcaacggaacttcctaatgcattccaattaaaac | C106S *entV* mutant |
| Vector_forward (promoter_dsbA) | tgaggtttttttatggatatttcagtaattgatgcaacaaaagttaacac | Gibson assembly for native *dsbA* promoter |
| Vector.Reverse (promoter_dsbA) | gttcacttttgcggccgctTTTTATTTGTTTTGAC | Gibson assembly for native *dsbA* promoter |
| Frag.Forward (promoter_dsbA) | Aagcggccgcaaaagtgaacagaggcacgaattc | Gibson assembly for native *dsbA* promoter |
| Frag.Reverse (promoter_dsbA) | ctgaaatatccataaaaaaacctcatcctttcaaacct | Gibson assembly for native *dsbA* promoter |
| SprEF2003 | GTTCCTGCCGAAAGTCATTC | *sprE* amplification for pTEX4577 |
| SprER2003 | GAAGTGAATTATAACTTTATTGT | *sprE* amplification for pTEX4577 |
